# End-to-End Formal Verification of Ethereum 2.0 Deposit Smart Contract

**DOI:** 10.1007/978-3-030-53288-8_8

**Published:** 2020-06-13

**Authors:** Daejun Park, Yi Zhang, Grigore Rosu

**Affiliations:** 8 Microsoft Research Lab, Redmond, WA USA; 9 University of Southern California, Los Angeles, CA USA; 10 Runtime Verification, Inc., Urbana, IL USA; 11 University of Illinois at Urbana-Champaign, Urbana, IL USA

## Abstract

We report our experience in the formal verification of the deposit smart contract, whose correctness is critical for the security of Ethereum 2.0, a new Proof-of-Stake protocol for the Ethereum blockchain. The deposit contract implements an incremental Merkle tree algorithm whose correctness is highly nontrivial, and had not been proved before. We have verified the correctness of the compiled bytecode of the deposit contract to avoid the need to trust the underlying compiler. We found several critical issues of the deposit contract during the verification process, some of which were due to subtle hidden bugs of the compiler.

## Introduction

The deposit smart contract [14] is a gateway to join Ethereum 2.0 [15] that is a new sharded Proof-of-Stake (PoS) protocol which at its early stage, lives in parallel with the existing Proof-of-Work (PoW) chain, called Ethereum 1.x chain. Validators drive the entire PoS chain, called Beacon chain, of Ethereum 2.0. To be a validator, one needs to deposit a certain amount of Ether, as a “stake”, by sending a transaction (over the Ethereum 1.x network) to the deposit contract. The deposit contract records the history of deposits, and locks all the deposits in the Ethereum 1.x chain, which can be later claimed at the Beacon chain of Ethereum 2.0.^1^ Note that the deposit contract is a one-way function; one can move her funds from Ethereum 1.x to Ethereum 2.0, but not vice versa.

The deposit contract, written in Vyper [19], employs the Merkle tree [30] data structure to efficiently store the deposit history, where the tree is *dynamically* updated (i.e., leaf nodes are incrementally added in order from left to right) whenever a new deposit is received. The Merkle tree employed in this contract is very large: it has height 32, so it can store up to $2^{32}$ deposits. Since the size of the Merkle tree is huge, it is not practical to reconstruct the whole tree every time a new deposit is received.

To reduce both time and space complexity, thus saving the gas^2^ cost significantly, the contract implements an *incremental Merkle tree algorithm* [6]. The incremental algorithm enjoys *O*(*h*) time and space complexity to reconstruct (more precisely, compute the root of) a Merkle tree of height *h*, while a naive algorithm would require $O(2^h)$ time or space complexity. The efficient incremental algorithm, however, leads to the deposit contract implementation being unintuitive, and makes it non-trivial to ensure its correctness. The correctness of the deposit contract, however, is critical for the security of Ethereum 2.0, since it is a gateway for becoming a validator. Considering the utmost importance of the deposit contract for the Ethereum blockchain, formal verification is demanded to ultimately guarantee its correctness.

In this paper, we present our formal verification of the deposit contract.^3^ The scope of verification is to ensure the correctness of the contract bytecode within a single transaction, without considering transaction-level or off-chain behaviors. We take the compiled bytecode as the verification target to avoid the need to trust the compiler.^4^

We adopt a refinement-based verification approach. Specifically, our verification effort consists of the following two tasks:

- Verify that the incremental Merkle tree algorithm implemented in the deposit contract is *correct* w.r.t. the original full-construction algorithm.
- Verify that the compiled bytecode is *correctly generated* from the source code of the deposit contract.

Intuitively, the first task amounts to ensuring the correctness of the contract source code, while the second task amounts to ensuring the compiled bytecode being a sound refinement of the source code (i.e., translation validation of the compiler). This refinement-based approach allows us to avoid reasoning about the complex algorithmic details, especially specifying and verifying loop invariants, directly at the bytecode level. This separation of concerns helped us to save a significant amount of verification effort. See Sect. 1.1 for more details.

*Challenges.* Formally verifying the deposit contract was challenging. First, the algorithm employed in the contract is sophisticated and its correctness is not straightforward to prove. Indeed, we found a critical bug in the algorithm implementation which had been not detected by existing tests (Sect. 3.1).

Second, we had to take the compiled bytecode as the verification target, which is much larger (consisting of $\sim$3,000 instructions) and more complex than the source code. The source-code-level verification was not accepted by the customer for the end-to-end correctness guarantee, especially considering the fact that the compiler is not mature enough [11]. Indeed, we found several new critical bugs in the compiler during the formal verification process (Sect. 3.2).

Third, we had to consider not only the functional correctness, but also security properties of the contract. That is, we had to identify the behaviors of the contract in exceptional cases, and check if they are exploitable. We found a bug of the contract in case that it receives invalid inputs (Sect. 3.3).

Finally, we had to take into account potential future changes in the Ethereum blockchain system (called hard-forks). That is, we had to verify that the compiled bytecode will work not only in the current system, but also in any future version of the system that employs a different gas fee schedule. Considering such potential changes of the system required us to generalize the semantics of bytecode execution. We also found a bug regarding that (Sect. 3.4).

### Our Refinement-Based Verification Approach

We illustrate our refinement-based formal verification approach used in the deposit contract verification. We present our approach using the K framework and its verification infrastructure [46, 52, 55], but it can be applied to other program verification frameworks.

Let us consider a $\texttt {sum}$ program that computes the summation from 1 to *n*: 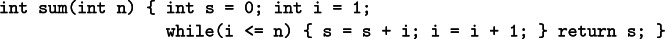


Given this program, we first manually write an abstract model of the program in the K framework [52]. Such a K model is essentially a state transition system of the program, and can be written as follows:
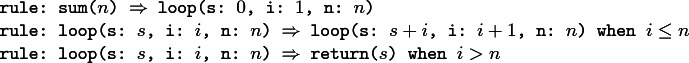


These transition rules correspond to the initialization, the $\texttt {while}$ loop, and the return statement, respectively. The indexed tuple $\texttt {(s:}\,s, \,\mathtt{i:}\,i, \,\mathtt{n:}\,n\mathtt{)}$ represents the state of the program variables $\texttt {s}$, $\texttt {i}$, and $\texttt {n}$.^5^

Then, given the abstract model, we specify the functional correctness property in reachability logic [54], as follows: 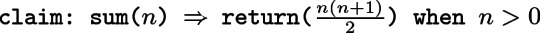


This reachability claim says that $\texttt {sum(}$*n*) will eventually return $\frac{n(n+1)}{2}$ in all possible execution paths, if *n* is positive. We verify this specification using the K reachability logic theorem prover [55], which requires us only to provide the following loop invariant:^6^ 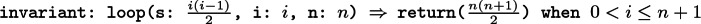


Once we prove the desired property of the abstract model, we manually refine the model to a bytecode specification, by translating each transition rule of the abstract model into a reachability claim at the bytecode level, as follows:
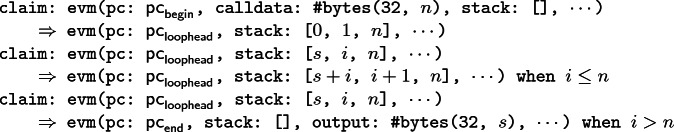


Here, the indexed tuple $\texttt {evm(pc:\_, calldata:\_, stack:\_, output:\_)}$ represents (part of) the Ethereum Virtual Machine (EVM) state, and $\texttt {\#bytes(}N,V\mathtt{)}$ denotes a sequence of *N* bytes of the two’s complement representation of *V*.

We verify this bytecode specification against the compiled bytecode using the same K reachability theorem prover [46, 55]. Note that no loop invariant is needed in this bytecode verification, since each reachability claim involves only a bounded number of execution steps—specifically, the second claim involves only a single iteration of the loop.

Then, we manually prove the soundness of the refinement, which can be stated as follows: *for any EVM states* $\sigma _1$ *and* $\sigma _2$, *if* $\sigma _1 \Rightarrow \sigma _2$, *then* $\alpha (\sigma _1) \Rightarrow \alpha (\sigma _2)$, where the abstraction function $\alpha$ is defined as follows:
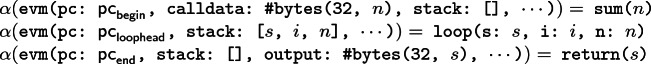


Putting all the results together, we finally conclude that the compiled bytecode will return $\texttt {\#bytes(32,}\frac{n(n+1)}{2}{} \mathtt{)}$.

Note that the abstract model and the compiler are *not* in the trust base, thanks to the refinement, while the K reachability logic theorem prover [46, 55] and the formal semantics of EVM [24] are.

## Formal Verification of the Deposit Contract

Following the refinement-based approach illustrated in Sect. 1.1, we first formalized the main business logic of the deposit contract (i.e., the incremental Merkle tree algorithm), and proved its correctness. Then we refined the formal model into a bytecode specification, and verified the compiled bytecode of the deposit contract against the refined specification. From these, we concluded the correctness of the deposit contract bytecode.

### Incremental Merkle Tree Algorithm

We briefly describe the incremental Merkle tree algorithm of the deposit contract. Due to space limitations, we omit the formalization of the algorithm and the formal proof of the correctness, and refer the readers to our companion technical report [45] for the full details.

**Fig. 1.**
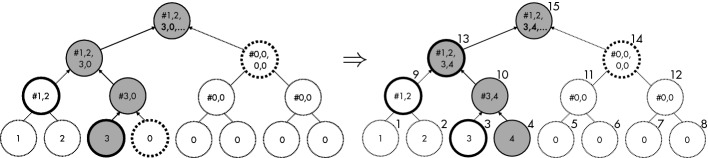
Illustration of the incremental Merkle tree algorithm. Node numbers are labeled in the upper-right corner of each node.

A Merkle tree [30] is a perfect binary tree [34] where leaf nodes store the hash of data, and non-leaf nodes store the hash of their children. A *partial Merkle tree up-to m* is a Merkle tree whose first (leftmost) *m* leaves are filled with data hashes and the other leaves are empty and filled with zeros. The incremental Merkle tree algorithm takes as input a partial Merkle tree up-to *m* and a new data hash, and inserts the new data hash into the $(m+1)^\text {th}$ leaf, resulting in a partial Merkle tree up-to $m+1$.

Figure 1 illustrates the algorithm, showing how the given partial Merkle tree up-to 3 (shown in the left) is updated to the resulting partial Merkle tree up-to 4 (in the right) when a new data hash is inserted into the ${4}^{\mathrm {th}}$ leaf node. The key idea of the algorithm is that only the path from the new leaf to the root (i.e., the gray nodes) needs to be computed (hence linear-time), and moreover the path can be computed by using only the left (i.e., node 3 and node 9) or right (i.e., node 14) sibling of each node in the path, which are only nodes that the algorithm maintains (hence linear-space). Refer to [45] for the full details.

### Bytecode Verification of the Deposit Contract

Now we present the formal verification of the compiled bytecode of the deposit contract. The bytecode verification ensures that the compiled bytecode is a sound refinement of the source code. This rules out the need to trust the compiler.

As illustrated in Sect. 1.1, we first manually refined the abstract model (in which we proved the algorithm correctness) to the bytecode specification. For the refinement, we consulted the ABI interface standard [13] (to identify, e.g., $\texttt {calldata}$ and $\texttt {output}$ in the illustrating example of Sect. 1.1), as well as the bytecode (to identify, e.g., the $\texttt {pc}$ and $\texttt {stack}$ information).^7^ Then, we used the KEVM verifier [46] to verify the compiled bytecode against the refined specification. We adopted the KEVM verifier to reason about all possible corner-case behaviors of the compiled bytecode, especially those introduced by certain unintuitive and questionable aspects of the underlying Ethereum Virtual Machine (EVM) [60]. This was possible because the KEVM verifier is derived from a complete formal semantics of the EVM, called KEVM [24]. Our formal specification and verification artifacts are publicly available at [50].

Let us elaborate on specific low-level behaviors verified against the bytecode. In addition to executing the incremental Merkle tree algorithm, most of the functions perform certain additional low-level tasks, and we verified that such tasks are correctly performed. Specifically, for example, given deposit data,^8^ the $\texttt {deposit}$ function computes its 32-byte hash (called Merkleization) according to the SimpleSerialize (SSZ) specification [18]. The leaves of the Merkle tree store only the computed hashes instead of the original deposit data. The $\texttt {deposit}$ function also emits a $\texttt {DepositEvent}$ log that contains the original deposit data, where the log message needs to be encoded as a byte sequence following the contract event ABI specification [13]. Other low-level operations performed by those functions that we verified include: correct zero-padding for the 32-byte alignment, correct conversions from big-endian to little-endian, input bytes of the SHA2-256 hash function being correctly constructed, and return values being correctly serialized to byte sequences according to the ABI specification [13].

We also verified a liveness property that the contract is always able to accept a new (valid) deposit as long as a sufficient amount of gas is provided. This liveness is not trivial since it needs to hold even in any future hard-fork where the gas fee schedule is changed. Indeed, we found a bug that violates the liveness. See Sect. 3.4 for more details.

Our formal specification includes both positive and negative behaviors. The positive behaviors describe the desired behaviors of the contracts in a legitimate input state. The negative behaviors, on the other hand, describe how the contracts handle exceptional cases (e.g., when benign users feed invalid inputs by mistake, or malicious users feed crafted inputs to take advantage of the contracts). The negative behaviors are mostly related to security properties.

For the full specification of the verified bytecode behaviors, refer to [49].

## Findings and Lessons Learned

In the course of our formal verification effort, we found subtle bugs [35–37] of the deposit contract, as well as a couple of refactoring suggestions [38–40] that can improve the code readability and reduce the gas cost. The subtle bugs of the deposit contract are partly due to bugs of the Vyper compiler [41–44] that we newly found (and reported to the Vyper team) in the verification process.

Below we elaborate on the bugs we found and lessons we learned along the way. We note that all the bugs of the deposit contract have been reported, confirmed, and properly fixed in the latest version (v0.11.2).

### Maximum Number of Deposits

In the original version of the contract that we were asked to verify, a bug is triggered when all of the leaf nodes of a Merkle tree are filled with deposit data, in which case the contract (specifically, the $\texttt {get\_deposit\_root}$ function) incorrectly computes the root hash of a tree, returning the zero root hash (i.e., the root hash of an empty Merkle tree) regardless of the content of leaf nodes. For example, suppose that we have a Merkle tree of height 2, which has four leaf nodes, and every leaf node is filled with certain deposit data, say $v_1$, $v_2$, $v_3$, and $v_4$, respectively. Then, while the correct root hash of the tree is $\mathsf {hash}(\mathsf {hash}(v_1,v_2),\mathsf {hash}(v_3,v_4))$, the $\texttt {get\_deposit\_root}$ function returns $\mathsf {hash}(\mathsf {hash}(0,0),\mathsf {hash}(0,0))$, which is incorrect.

Due to the complex logic of the code, it is non-trivial to properly fix this bug without significantly rewriting the code, and thus we suggested a workaround that simply forces to never fill the last leaf node, i.e., accepting only $2^h - 1$ deposits at most, where *h* is the height of a tree. We note that, however, it is infeasible in practice to trigger this buggy behavior in the current setting, since the minimum deposit amount is 1 Ether and the total supply of Ether is less than 130M which is much smaller than $2^{32}$, thus it is not feasible to fill all the leaves of a tree of height 32. Nevertheless, this bug has been fixed by the contract developers as we suggested, since the contract may be used in other settings in which the buggy behavior can be triggered and an exploit may be possible. Refer to [37] for more details.

We also want to note that this bug was quite subtle to catch. Indeed, we had initially thought that the original code was correct until we failed to write a formal proof of the correctness theorem. The failure of our initial attempt to prove the correctness led us to identify a missing premise that was needed for the theorem to hold, from which we could find the buggy behavior scenario, and suggested the bugfix. This experience reconfirms the importance of formal verification. Although we were not “lucky” to find this bug when we had eyeball-reviewed the code, which is all traditional security auditors do, the formal verification process thoroughly guided and even “forced” us to find it eventually.

### ABI Standard Conformance of $\texttt {get\_deposit\_count}$ Function

In the previous version, the $\texttt {get\_deposit\_count}$ function does not conform to the ABI standard [13], where its return value contains incorrect zero-padding [35], due to a Vyper compiler bug [41]. Specifically, in the buggy version of the compiled bytecode, the $\texttt {get\_deposit\_count}$ function, whose return type is $\texttt {bytes[8]}$, returns a byte sequence of length 96, where the last byte is $\texttt {0x20}$ while it should be $\texttt {0x00}$. According to the ABI specification [13], the last 24 bytes must be all zero, serving as zero-pad for the 32-byte alignment. Thus the return value does not conform to the ABI standard. This is problematic because any contract (written in either Solidity or Vyper) that calls to (the buggy version of) the deposit contract, expecting that the $\texttt {deposit\_count}$ function conforms to the ABI standard, could have misbehaved.^9^

This buggy behavior is mainly due to a subtle Vyper compiler bug [41] that fails to correctly compile a function whose return type is $\texttt {bytes[}n\mathtt{]}$ where $n < 16$. This leads to the compiled function returning a byte sequence with insufficient zero-padding as mentioned above, failing to conform to the ABI standard.

We note that this bug could not have been detected if we did not take the bytecode as the verification target. This reconfirms that the bytecode-level verification is critical to ensure the ultimate correctness (unless we formally verify the underlying compiler), because we cannot (and should not) trust the compiler.

### Checking Well-Formedness of Calldata

The calldata decoding process in the previous version of the compiled bytecode does not have sufficient runtime-checks for the well-formedness of calldata. As such, it fails to detect certain ill-formed calldata, causing invalid deposit data to be put into the Merkle tree. This is problematic especially when clients make mistakes and send deposit transactions with incorrectly encoded calldata, which may result in losing their deposit fund.

Specifically, we found a counter-example ill-formed calldata whose size (196 bytes) is much less than that of well-formed calldata (356 bytes). The problem, however, is that the $\texttt {deposit}$ function does *not* reject the ill-formed calldata, but simply inserts certain invalid (garbage) deposit data in the Merkle tree. Since the invalid deposit data cannot pass the signature validation later, no one can claim the deposited fund associated with this, and the deposit owner loses the fund. Note that this happens even though the $\texttt {deposit}$ function employs assertions at the beginning of the function that ensures the size of each of the arguments is correct, which turned out to not work as expected.

This problem would not exist if the Vyper compiler thoroughly generated runtime checks to ensure the well-formedness of calldata.^10^ However, since it was not trivial to fix the compiler to generate such runtime checks, we suggested several ways to improve the deposit contract source code to prevent this behavior without fixing the compiler. After careful discussion with the deposit contract development team, we together decided to employ a checksum-based approach where the $\texttt {deposit}$ function takes as an additional input a checksum for the deposit data, and rejects any ill-formed calldata using the checksum. The checksum-based approach is the least intrusive and the most gas-efficient of all the suggested fixes. For more details of other suggested fixes, refer to [36].

We note that this issue was found when we were verifying the negative behaviors of the deposit contract. This shows the importance of having the formal specification to include not only positive but also negative behaviors.

### Liveness

As mentioned in Sect. 2.2, the previous version of the deposit contract fails to satisfy a liveness property in that it may not be able to accept a new deposit, even if it is valid, in a certain future hard-fork that updates the gas fee schedule. This was mainly due to another subtle Vyper compiler bug [44] that generates bytecode where a hard-coded amount of gas is supplied when calling to certain precompiled contracts. Although this hard-coded amount of gas is sufficient in the current hard-fork (code-named Istanbul [17]), it may not be sufficient in a certain future hard-fork that increases the gas fee schedule of the precompiled contracts. In such a future hard-fork, the previous version of the deposit contract will always fail due to the out-of-gas exception, regardless of how much gas is initially supplied. Refer to [44] for more details.

We admit that we could not find this issue until the deposit contract development team carefully reviewed and discussed with us the formal specification [49] of the bytecode. Initially, we considered only the behaviors of the bytecode in the current hard-fork, without identifying the requirement that the contract bytecode should work in any future hard-fork. We identified the missing requirement, and found this liveness issue, at a very late stage of the formal verification process, which delayed the completion of formal verification.

This experience essentially illustrates the well-known problem caused by the gap between the intended behaviors (that typically exists only informally) by developers, and the formal specification written by verification engineers. To reduce this gap, the two groups should work closely together, or ideally, developers should write their own specifications in the first place. For the former, the formal verification process should involve developers more frequently. For the latter, the formal verification tools should become much easier to use without requiring advanced knowledge of formal methods. We leave both as future work.

### Discussion

*Verification Effort.* The net effort for formal verification took 7 person-weeks (excluding various discussions with developers, reporting bugs and following-up, especially for compiler bugs, etc.), where the algorithm correctness proof took 2 person-weeks, and the bytecode verification took 5 person-weeks. This includes the time spent on writing specifications as well. The bytecode specification consists of $\sim$1,000 LOC (excluding comments), in addition to auxiliary lemmas consisting of $\sim$200 LOC. The size of the source code is $\sim$100 LOC, and the number of instructions in the compiled bytecode is $\sim$3,000.

*Trust Base.* The validity of the bytecode verification result assumes the correctness of the bytecode specification and the KEVM verifier. The algorithm correctness proof is partially mechanized—only the proof of major lemmas are mechanized in the K framework. The non-mechanized proofs are included in our trust base. The Vyper compiler is *not* in the trust base.

*Continuous Verification.* The verification target contract was a moving target. Even if the contract code had been frozen before starting the formal verification process, the code (both source code or bytecode) was updated in the middle of the verification process, to fix bugs found during the process. Indeed, we found several bugs in both the contract and the compiler, and each time we found a bug, we had to re-verify the newly compiled bytecode that fixes the bug. Here the problem was the overhead of re-verification. About 20% of the bytecode verification effort was spent on re-verification.

The re-verification overhead could have been reduced by automatically adjusting formal specifications to updated bytecode, and/or making specifications as independent of the specific details of the bytecode as possible. For example, the current bytecode specification employs specific program-counter (PC) values to refer to some specific positions of the bytecode, especially when specifying loop invariants. Most of such PC values need to be updated whenever the bytecode is modified. The re-verification overhead could have been reduced by automatically updating such PC values, or even having the specification refer to specific positions without using PC values. We leave this as future work.

## Related Work

*Static Analysis and Verification of Smart Contracts.* There have been proposed many static analysis tools [5, 10, 20, 25, 28, 29, 32, 57, 58] that are designed to automatically detect a certain fixed set of bugs and vulnerabilities of smart contracts, at the cost of generality and expressiveness. VerX [47] can verify past-time linear temporal properties over multiple runs of smart contracts, but it requires the target contracts to be effectively loop-free.

There also have been proposed verification tools that allow us to specify and verify arbitrary functional correctness and/or security properties, such as [3, 22] based on the F* proof assistant [1, 56] based on Isabelle/HOL [33], the KEVM verifier [46] based on the K framework [52], and VeriSol [27] based on Boogie [2]. The KEVM verifier has also been used to verify high-profile and challenging smart contracts [51], including a multi-signature wallet called Gnosis Safe [21], a decentralized token exchange called Uniswap [59], and a partial consensus mechanism called Casper FFG [7].

*Verification of Systems Software.* There are many success stories of formal verification of systems software, from OS kernels [23, 26, 31], to file systems [8, 53], to cryptographic code [4]. While most of the verified systems code is either synthesized from specifications, or implemented (or adjusted) to be verification-friendly, there also exist efforts [9, 12] to verify actual production code as is. Such efforts are necessary especially when the production code is highly performance-critical and/or existing development processes are hard to change to help produce verification-friendly code. The deposit contract we verified was given to us at the code-frozen stage, and also performance-critical (especially in terms of the gas cost), and thus we took and verified the given production-ready code as is, without any modification except for fixing bugs.

## Footnotes

1 This deposit process will change at a later stage.

2 In Ethereum, gas refers to the fee to execute a transaction or a smart contract on the blockchain. The amount of gas fee depends on the size of the payloads.

3 This was done as part of a contract funded by the Ethereum Foundation [16].

4 Indeed, we found several new critical bugs [41–44] of the Vyper compiler in the process of formal verification. See Sect. 3 for more details.

5 Note that this abstract model can be also automatically derived by instantiating the language semantics with the particular program, if a formal semantics of the language is available (in the K framework).

6 The loop invariants in reachability logic mentioned here look different from those in Hoare logic. See the comparison between the two logic proof systems in [55, Section 4]. These loop invariants can be also seen as transition invariants [48].

7 However, we want to note that the Vyper compiler can be augmented to extract such information, which can automate the refinement process to a certain extent. We leave that as future work.

8 Each deposit data consists of the public key, the withdrawal credentials, the deposit amount, and the signature of the deposit owner.

9 The returned byte sequence, including the incorrect last byte, is copied to the caller’s memory. If the caller reuses the last byte assuming that it is zero, the garbage value will be passed around, which may break the business logic of the caller.

10 The compiler developers failed to consider the case when the given calldata is not correctly encoded. For example, while the header of calldata contains offsets (i.e., pointers) to the positions of data elements, it could be the case that certain offsets are beyond the calldata range. In that case, the calldata can be accessed outside its bounds, due to the missing runtime-checks.
